# The long-term survival and functional maturation of human iNPC-derived neurons in the basal forebrain of cynomolgus monkeys

**DOI:** 10.1093/lifemedi/lnac008

**Published:** 2022-06-28

**Authors:** Su Feng, Ting Zhang, Wei Ke, Yujie Xiao, Zhong Guo, Chunling Lu, Shuntang Li, Zhongxin Guo, Yuanyuan Liu, Guohe Tan, Yingying Chen, Feng Yue, Yousheng Shu, Chunmei Yue, Naihe Jing

**Affiliations:** Bioland Laboratory/Guangzhou Laboratory, Guangzhou 510005, China; State Key Laboratory of Cell Biology, CAS Center for Excellence in Molecular Cell Science, Shanghai Institute of Biochemistry and Cell Biology, Chinese Academy of Sciences; University of Chinese Academy of Sciences, Shanghai 200031, China; State Key Laboratory of Cell Biology, CAS Center for Excellence in Molecular Cell Science, Shanghai Institute of Biochemistry and Cell Biology, Chinese Academy of Sciences; University of Chinese Academy of Sciences, Shanghai 200031, China; Department of Ophthalmology, Shanghai General Hospital, Shanghai Jiao Tong University, School of Medicine, Shanghai Key Laboratory of Ocular Fundus Diseases, Shanghai Engineering Center for Visual Science and Photomedicine, Shanghai 200080, China; National Clinical Research Center for Ophthalmic Diseases, Shanghai 200080, China; Department of Neurology, Huashan Hospital, State Key Laboratory of Medical Neurobiology, Institutes for Translational Brain Research, MOE Frontiers Center for Brain Science, Fudan University, Shanghai 200032, China; Department of Neurology, Huashan Hospital, State Key Laboratory of Medical Neurobiology, Institutes for Translational Brain Research, MOE Frontiers Center for Brain Science, Fudan University, Shanghai 200032, China; Wincon TheraCells Biotechnologies Co, LTD, Nanning 530000, China; Wincon TheraCells Biotechnologies Co, LTD, Nanning 530000, China; Guangxi Key Laboratory of Regenerative Medicine and Key Laboratory of Longevity and Aging-related Diseases of Chinese Ministry of Education, Collaborative Innovation Centre of Regenerative Medicine and Medical BioResource Development and Application Co-constructed by the Province and Ministry, School of Basic Medical Sciences, Guangxi Medical University, Nanning 530021, China; Guangxi Key Laboratory of Regenerative Medicine and Key Laboratory of Longevity and Aging-related Diseases of Chinese Ministry of Education, Collaborative Innovation Centre of Regenerative Medicine and Medical BioResource Development and Application Co-constructed by the Province and Ministry, School of Basic Medical Sciences, Guangxi Medical University, Nanning 530021, China; Guangxi Key Laboratory of Regenerative Medicine and Key Laboratory of Longevity and Aging-related Diseases of Chinese Ministry of Education, Collaborative Innovation Centre of Regenerative Medicine and Medical BioResource Development and Application Co-constructed by the Province and Ministry, School of Basic Medical Sciences, Guangxi Medical University, Nanning 530021, China; Guangxi Key Laboratory of Regenerative Medicine and Key Laboratory of Longevity and Aging-related Diseases of Chinese Ministry of Education, Collaborative Innovation Centre of Regenerative Medicine and Medical BioResource Development and Application Co-constructed by the Province and Ministry, School of Basic Medical Sciences, Guangxi Medical University, Nanning 530021, China; State Key Laboratory of Cell Biology, CAS Center for Excellence in Molecular Cell Science, Shanghai Institute of Biochemistry and Cell Biology, Chinese Academy of Sciences; University of Chinese Academy of Sciences, Shanghai 200031, China; Wincon TheraCells Biotechnologies Co, LTD, Nanning 530000, China; School of Biomedical Engineering, Hainan University, Haikou 570228, China; Department of Neurology, Huashan Hospital, State Key Laboratory of Medical Neurobiology, Institutes for Translational Brain Research, MOE Frontiers Center for Brain Science, Fudan University, Shanghai 200032, China; State Key Laboratory of Cell Biology, CAS Center for Excellence in Molecular Cell Science, Shanghai Institute of Biochemistry and Cell Biology, Chinese Academy of Sciences; University of Chinese Academy of Sciences, Shanghai 200031, China; Department of Biological Sciences, School of Science, Xi’an Jiaotong-Liverpool University, Suzhou 215000, China; Bioland Laboratory/Guangzhou Laboratory, Guangzhou 510005, China; State Key Laboratory of Cell Biology, CAS Center for Excellence in Molecular Cell Science, Shanghai Institute of Biochemistry and Cell Biology, Chinese Academy of Sciences; University of Chinese Academy of Sciences, Shanghai 200031, China; CAS Key Laboratory of Regenerative Biology, Guangdong Provincial Key Laboratory of Stem Cell and Regenerative Medicine, Guangzhou Institutes of Biomedicine and Health, Chinese Academy of Sciences, Guangzhou 510530, China; Institute for Stem Cell and Regeneration, Chinese Academy of Sciences, Beijing 100101, China

**Keywords:** non-human primates, cerebral cell transplantation, human induced neural stem/progenitor cells, neuronal differentiation, functional maturation

## Abstract

Human induced neural stem/progenitor cells (iNPCs) are a promising source of cells for stem cell-based therapy. The therapeutic potential of human iNPCs has been extensively tested in animal models, including both mouse and monkey models. However, the comprehensive characterization of grafted iNPCs in the brain of non-human primates has been lagged behind. In this study, we transplanted human iNPCs into the basal forebrain of adult cynomolgus monkeys. We found that grafted iNPCs predominantly differentiated into neurons that displayed long-term survival up to 12 months. Additionally, iNPC-derived human neurons gradually matured in term of morphology and subtype differentiation. More excitingly, we observed that human neurons displayed electrophysiological activities resembling those of mature neurons, indicating the acquisition of functional membrane properties. Collectively, this study systematically characterized human iNPCs in the brain of non-human primates, and will provide invaluable clues for developing safe and effective stem cell-based therapies for different brain disorders.

## Introduction

With the advancement of reprogramming technology, induced neural stem/progenitor cells (iNPCs) can be directly produced from human somatic cells by ectopic expression of transcription factors and treatment of small molecules [1–4]. Resembling native NPCs, human iNPCs are self-renewable and multipotent cells that can give rise to neurons and astrocytes [5, 6]. Compared with native NPCs, human iNPCs are expandable *in vitro* and easily accessible. Moreover, human iNPCs exhibit high homogeneity, express little if any pluripotent marker OCT4 and show minus tumorigenic potential compared to NPCs derived from pluripotent stem cells, mainly from embryonic stem cells or iPSCs [7, 8]. Therefore, human iNPCs possess distinct advantages as donor cells in cell therapies with clinical potential.

Previously, the therapeutic potential of human NPCs, including iNPCs, has been assessed in mouse models of different brain disorders, such as Parkinson’s disease (PD) and Alzheimer’s disease (AD) [3, 9, 10]. In recent years, attempts have been made to investigate the therapeutic benefits of human iNPCs in monkeys, mainly in lesioned monkey models of PD [11, 12]. Due to the high similarities between non-human primates and humans, cell transplantation studies in monkeys are believed to provide more informative clues for designing clinical trials for cell therapies [13]. It turns out that the engraftment of human iNPCs leads to evident restoration of behavioral deficits associated with PD or AD in both mouse and monkey models, confirming the therapeutical promise of iNPC-based cell therapies for brain disorders [14]. However, the cellular mechanisms underpinning these repairment processes remain largely undetected, particularly in non-human primates.

Our previous studies have examined the survival, differentiation, maturation, migration, projection and functional integration of human iNPCs in the brain of AD mouse, elucidating cellular events underlying the functional recovery of host animals due to cell transplantation [3, 15]. It is critical but difficult to evaluate the maturation and integration of grafts in the host brain by measuring the electrophysiological activities of neurons derived from iNPCs. If the grafted cells mature and function as neurons, they should receive synaptic inputs and generate the main neuronal outputs, such as the APs. It is better to examine these electrophysiological properties *in situ* than in culture systems. However, due to the technical challenges, previous electrophysiological measurements have been limited on neurons cultured in the dish or transplanted in young rodents [16, 17]. The investigations of electrical activities of grafted human iNPCs in the brain of non-human primates have been rare. In this follow-up study, we performed systematic characterization of grafted human iNPCs in adult monkey brains and observed the long-term survival, predominant neuronal differentiation of human iNPCs. More importantly, the patch-clamp recording in adult monkey brain slices was carried out and identified electrophysiological properties that human iNPC-derived neurons possessed. We thereby revealed the functional maturation of human neurons derived from grafted iNPCs in the monkey brain.

## Results

### Human iNPCs differentiated into neurons displaying long-term survival in monkey brains

To test human NPCs in the brain of non-human primates, the homogeneous human iNPCs that were derived from immobilized adult peripheral blood mononuclear cells were used for transplantation in this study [3]. The previous study revealed that these human iNPCs resembled native human NPCs and successfully served as donor cells in AD mouse [3]. We recruited five adult cynomolgus monkeys that were around 11 years old from the same colony (Table 1). The MRI-guided stereotaxic injections were performed to bilaterally deliver GFP-labeled human iNPCs into the basal forebrain of monkeys. In total, 8 × 10^6^ cells were delivered into the basal forebrain of each monkey via 4 injections (2 injections/side). Characterization of survival and differentiation of grafted human iNPCs was carried out in 3 of 5 monkeys at three different time points, and electrophysiological measurements were performed in the rest two monkeys 10 months post transplantation (Table 1).

**Table 1.** The information of monkeys for transplantation

Monkey ID	Age (years)	Sex	Injection site	Tracts	Total number of human iNPCs injected	Time p.t. (months)	GFP^ + ^grafts	Experiments performed
052002	11	F	Basal forebrain	4	8 × 10^6^	4	+	Immunostaining analysis
042208	12	F	Basal forebrain	4	8 × 10^6^	8	+	Immunostaining analysis
062226	10	F	Basal forebrain	4	8 × 10^6^	12	+	Immunostaining analysis
061502	12	M	Basal forebrain	4	8 × 10^6^	10	+	Electrophysiological measurement
071487	11	M	Basal forebrain	4	8 × 10^6^	10	+	Electrophysiological measurement

**Figure 1.**
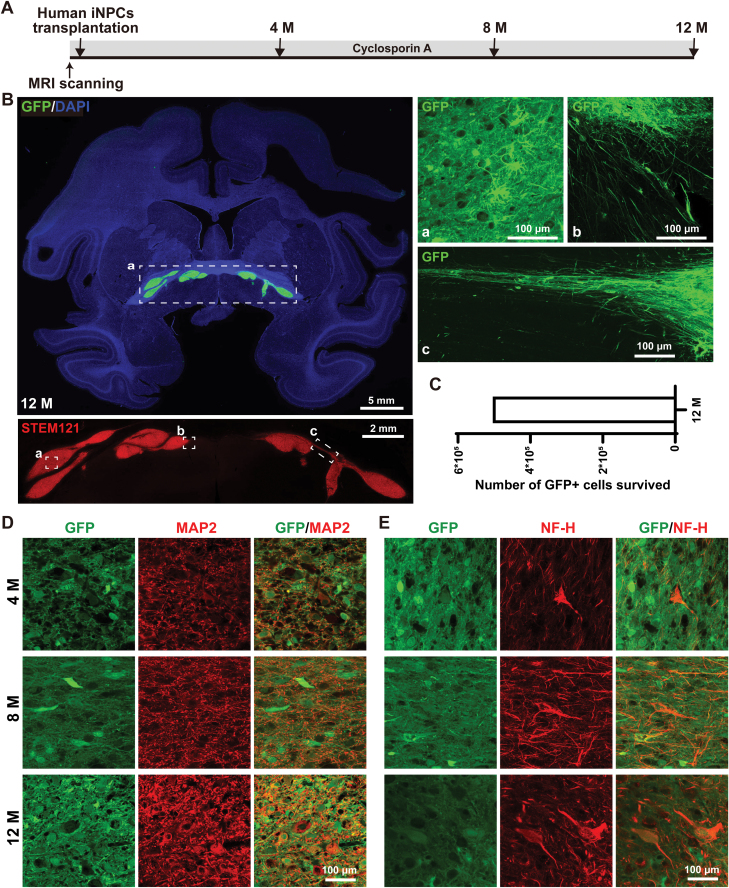
The survival and neuronal differentiation of human iNPCs in the basal forebrain of monkeys. (A) Schematic diagram of human iNPCs transplantation, immunosuppression treatment by cyclosporin A and time points to collect data. (B) Survival analysis of human grafts in the basal forebrain of a cynomolgus monkey 12 months post transplantation. Upper panel, the coronal image of whole monkey brain with GFP^ + ^grafts in the basal forebrain; lower panel, immunostaining for human-specific cytoplasmic marker STEM121; (a–c) the fibers from GFP^ + ^human cells. Cell nuclei were counterstained with DAPI. (C) The number of survived human GFP^ + ^cells in the basal forebrain of monkey 12 months post transplantation. (D, E) Immunofluorescence analysis of the expression of neuronal markers MAP2 and NF-H within human GFP ^+^ grafts 4, 8 and 12 months post transplantation. Scale bars: 5 mm in whole brain image in (B); 2 mm in enlarged view; 100 μm in enlarged views in (a–c); 100 μm in (D–F).

The survival and differentiation of grafted human iNPCs were assessed 4, 8 and 12 months post transplantation in three monkeys (Fig. 1A). Daily administration of cyclosporine A was given from 2 days before transplantation until the day of euthanasia for immunosuppression (Fig. 1A). We observed GFP^ + ^cells residing in the basal forebrain at each time point measured, as those grafts shown after 12 months in the monkey brain (Fig. 1B, left upper panel). Further measurements revealed that grafted GFP^ + ^cells were exogenous human cells expressing STEM121, a ­human-specific cytoplasmic marker (Fig. 1B, left lower panel). In addition, GFP^ + ^grafts projected long and abundant axon-like fibers, which mainly migrated along anterior commissure over a long distance (Fig. 1Ba–c). The total number of survived GFP^ + ^human cells within the monkey basal forebrain 12 months post transplantation was approximately 5 × 10^5^ (Fig. 1C), indicative of the long-term survival of human iNPCs in monkey brains. More importantly, the grafted human iNPCs differentiated into neurons expressing multiple neuronal markers, such as TUJ1 (Fig. S1), MAP2 (Fig. 1D) and neurofilament heavy chain (NF-H) (Fig. 1E) in all three time points.

Together, the above data indicate that grafted human iNPCs have survived well for a long time in the monkey brain and possessed the capability to differentiate into neurons.

### Human iNPCs gave rise to astrocytes and exhibited declined proliferative ability in monkey brains

**Figure 2.**
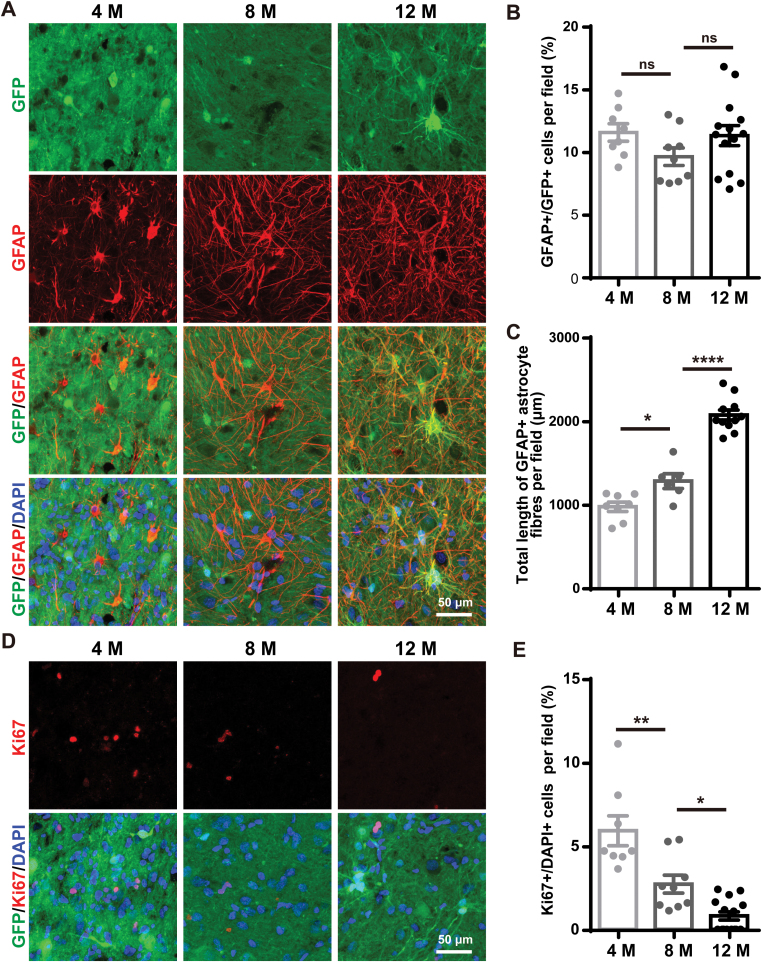
The astrocyte differentiation and proliferation of human iNPCs in the basal forebrain of monkeys. (A) Immunofluorescence analysis of GFAP expression within human GFP^ + ^grafts 4, 8 and 12 months post transplantation. Cell nuclei were counterstained with DAPI. (B) Percentages of GFAP^ + ^astrocytes among GFP^ + ^grafted human cells shown in (A). (C) Quantification of total length of fibers from GFAP^ + ^astrocytes shown in (A). (D) Immunofluorescence analysis of Ki67 expression within human GFP^ + ^grafts 4, 8 and 12 months post transplantation. Cell nuclei were counterstained with DAPI. (E) Percentages of Ki67^ + ^proliferating cells among total cells within GFP^ + ^grafts shown in (D). Scale bars: 50 μm in (A) and (D). Data are represented as scatterplots with mean ± SEM, One-way ANOVA with Tukey’s multiple comparison post hoc test. *P < .05, **P < .01, ****P < .0001, ns (not significant).

In addition to neurons, we also observed that grafted human iNPCs differentiated into GFAP^ + ^astrocytes in monkey brains (Fig. 2A). The percentages of astrocytes among grafted human cells were relatively consistent, which were approximately 10% at all three monkeys measured (Fig. 2B and Table S1). However, the detected astrocytes exhibited more complicated morphology with more and longer fibers in a time-dependent manner (Figs. 2A, 2C and Table S1). These observations indicated that the human iNPC-derived astrocytes were gradually matured over time in the host basal forebrain.

To address the safety concerns of human iNPCs in the monkey brain, we tested the proliferation of grafted cells by measuring the expression of Ki67 (Fig. 2D and Table S1). We found that approximately 6% of cells among grafts kept proliferating 4 months post transplantation, which dramatically decreased to 3% after 8 months and then 1% after 12 months (Figs. 2D, 2E and Table S1). Therefore, grafted human iNPCs displayed limited proliferative ability that rapidly lost in the monkey brain. It is an essential feature for human iNPCs as donor cells, which sustains the long-term survival and avoids graft overgrowths.

Collectively, these results show that grafted human iNPCs are multi-potential neural progenitors and give rise to both neurons and astrocytes in monkey brains.

### Human iNPC-derived neurons matured gradually in monkey brains

**Figure 3.**
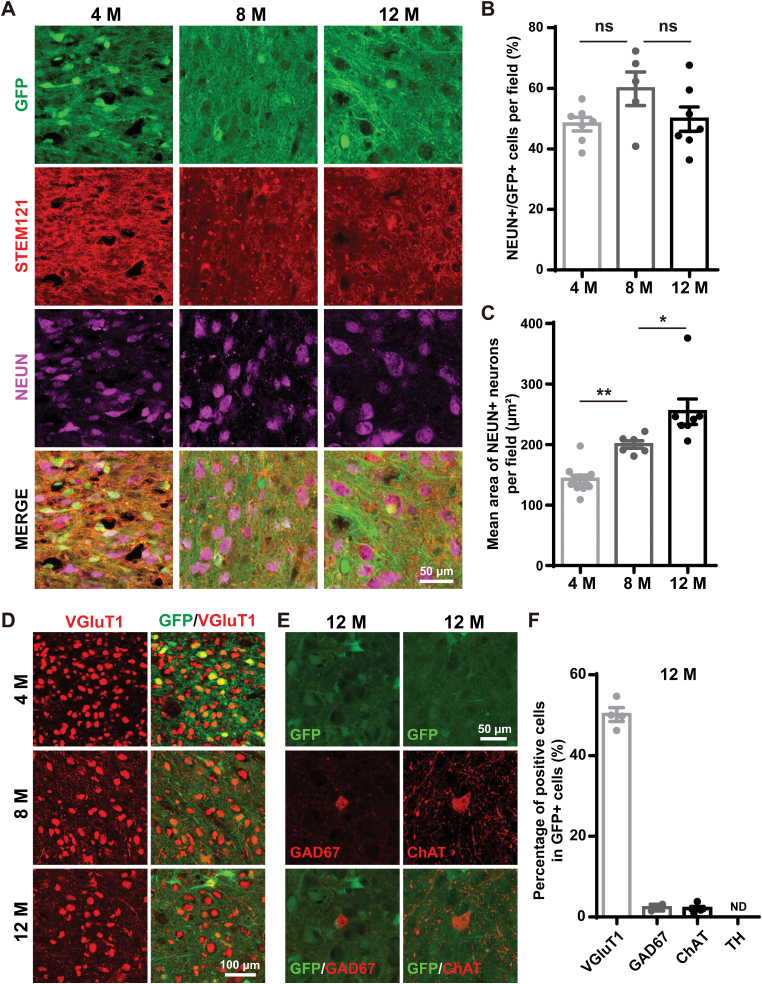
The maturation of human iNPC-derived neurons in the basal forebrain of monkeys. (A) Immunofluorescence analysis of STEM121 and NEUN expression within GFP^ + ^grafts 4, 8 and 12 months post transplantation. (B) Percentages of NEUN^ + ^neurons among GFP^ + ^grafted human cells shown in (A). (C) Quantification of mean area of nuclei of NEUN^ + ^neurons shown in (A). (D) Immunofluorescence analysis of VGluT1 expression within GFP^ + ^grafts 4, 8 and 12 months post transplantation. (E) Immunofluorescence analysis of GAD67 and ChAT expression within GFP^ + ^grafts 12 months post transplantation. (F) Percentages of different subtypes of neurons among GFP^ + ^grafted human cells 12 months post transplantation shown in (D) and (E). Scale bars: 50 μm in (A) and (E); 100 μm in (D). Data are represented as scatterplots with mean ± SEM, One-way ANOVA with Tukey’s multiple comparison post hoc test. *P < .05, **P < .01, ns (not significant).

To further investigate human iNPC-derived neurons in monkey brains, we performed double immunostaining in the grafts 4, 8 and 12 months after transplantation, and found that NEUN^ + ^neurons within grafted cells were STEM121^ + ^human neurons (Fig. 3A and Fig. S2). The percentages of human iNPC-derived neurons among grafted cells were relatively consistent in all three monkeys tested, which were around 50%–60% (Fig. 3B and Table S1). Compared with the percentage of iNPC-derived astrocytes, the transplanted human iNPCs mostly differentiated into neurons, and showing a clear bias of neuronal differentiation potency. In addition, we observed that NEUN^ + ^neurons became more scattered in the grafts (Fig. 3A), and the size of neuronal nuclei enlarged in a time-dependent manner (Fig. 3C), indicating that human iNPC-derived neurons gradually matured in monkey brains. Aside from morphology, the grafted human neurons further acquired distinct neuronal subtypes. We found that the predominant subtype of neurons was VGluT1^ + ^glutamatergic neurons, which accounted for at least 50% of grafted cells 12 months after transplantation (Figs. 3D and 3F). The percentages of GAD67^ + ^GABAergic neurons and ChAT^ + ^cholinergic neurons were less than 1%, and the TH^ + ^dopaminergic neurons were not detectable (Figs. 3E and 3F).

Together, these results clearly show the maturation of human iNPC-derived neurons in monkey brains.

### Human iNPC-derived neurons functioned well in monkey brains

**Figure 4.**
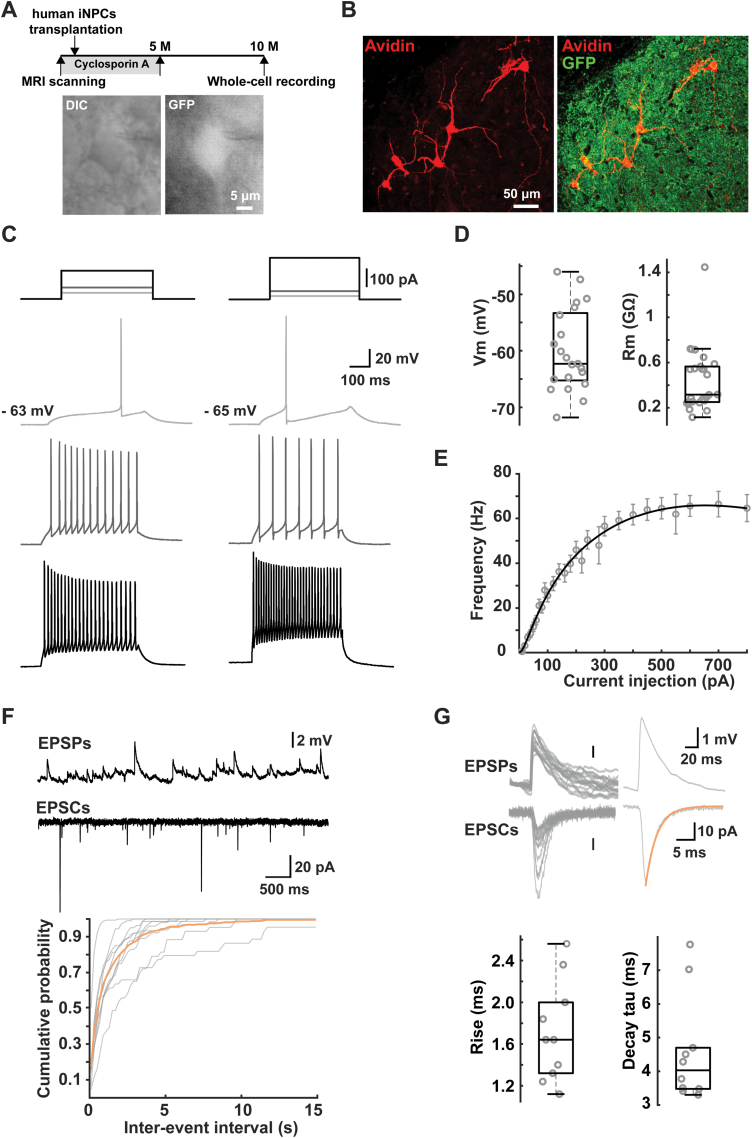
The electrophysiological activities of human iNPC-derived neurons in host basal forebrain and monkey granule cells. (A) Upper: experimental diagram of human iNPCs transplantation, immunosuppression treatment and electrophysiological measurements. Note that *in vitro* monkey brain slices were collected 10 months after hiNPCs transplantation, the cyclosporin A treatment for the first 5 months; lower: DIC and fluorescent images showing the identification of GFP-positive grafted cells. (B) The morphology of the recorded grafted cells showed by post hoc avidin staining. Note the neurites emitted from the recorded cells. (C) Repetitive firing induced by current steps shown in the top row. Each column represents one example cell. (D) Group data of the resting membrane potential and input resistance (E). (F–I) curve of the recorded grafted cells showing the dependence of firing frequency on current intensity. (F) Upper: example traces showing the occurrence of spontaneous EPSPs and EPSCs; lower: gray, cumulative probability of EPSCs inter-event interval for each cell; yellow, average cumulative probability. (G) Upper: aligned example EPSPs and EPSCs, and their averaged traces with the decay phase of EPSC fitted with an exponential function; lower: group data for 10%–90% rise time and decay time constant of EPSCs. Scale bars: 5 μm in (A); 50 μm in (B). Data are represented as scatterplots with mean ± SED.

To assess whether matured human neurons could function in the monkey brain, the electrophysiological measurements were performed on the rest two monkeys 10 months after transplantation (Table 1). These two monkeys were treated by cyclosporine A following a half-term immunosuppression strategy (Fig. 4A). Similarly, the transplanted human iNPCs were found to give rise to both neurons and astrocytes in host basal forebrain (Figs. S3A and S3B).

Acute slices harboring human grafts were made from monkey brains 10 months after transplantation. Whole-cell patch-clamp recording was performed from GFP-positive grafted cells in brain slices under a fluorescence microscope (Fig. 4A). Since the patch pipettes contained biocytin, the recorded cells could be visualized via post hoc avidin staining, allowing examination of their morphological features (Fig. 4B). We injected a series of step currents to examine the passive and active membrane properties. The grafted GFP-positive neurons had average input resistance of 440 MΩ (*n* = 26 cells) and resting membrane potential of ‐60.0 mV (*n* = 22 cells) (Fig. 4D). Among 27 recorded cells, only one cell produced single APs, regardless of the amount current injection, all other cells showed repetitive firing with increasing step currents, a characteristic feature of mature neurons (Fig. 4C). Accordingly, the *F*–*I* curve of the grafted human cells showed a gradual increase in action potential frequency with increasing current injections from 20 to 800 pA (Fig. 4E). At the resting membrane potential, we observed spontaneous excitatory postsynaptic potentials (sEPSPs) in recorded cells, indicative of the formation of functional synapses onto these human neural cells. In voltage-clamp mode at a holding potential of ‐70 mV, we observed barrages of synaptic currents (sEPSCs), with median frequency of 2.09 Hz (Fig. 4F). The sEPSCs had average rise time and decay time constant of 1.64 and 4.53 ms, respectively (*n* = 10 cells) (Fig. 4G), which were comparable to those of mature neurons in monkey prefrontal cortex [18]. Consistently, we observed that grafted human neurons extensively expressed presynaptic marker synaptophysin (SYP), and the presence of robust SYP^ + ^dots within GFP^ + ^grafts also implicates the formation of synapses (Fig. S2C). Collectively, these results demonstrated that human iNPC-derived neurons functionally matured and integrated into the synaptic network of host basal forebrain.

## Discussion

**Figure 5.**
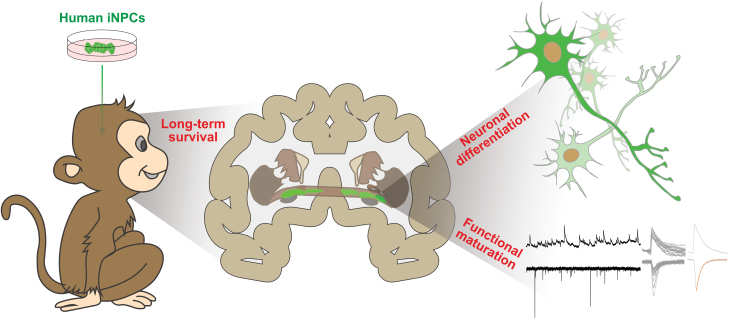
Graphical summary. The bilateral transplantation of GFP-labeled human iNPCs into the basal forebrain of monkeys (left panel), the long-term survival and neuronal differentiation of human iNPCs in the monkey brain (middle panel and right upper panel), and the functional maturation of human iNPC-derived neurons (right lower panel).

In this study, we transplanted human iNPCs into the basal forebrain of adult cynomolgus monkeys. Characterizations of grafts revealed the long-term survival and predominant neuronal differentiation of human iNPCs. More importantly, human ­iNPC-derived neurons gradually matured in the monkey brain and acquired functional membrane properties resembling mature neurons (Fig. 5).

Cell therapy is the central application of human neural stem/progenitor cells (NPCs). Extensive studies have focused on generating different types of human NPCs from fetal brain tissue, then from pluripotent stem cells, or from somatic cells recently [6, 19, 20]. Among them, human induced NPCs (iNPCs) derived from healthy somatic cells are considered as the proper donor cells with tremendous therapeutic potential in treating diverse neurological diseases, such as PD and AD. Meanwhile, the therapeutic potential of human NPCs has been widely assessed and confirmed in animal models of different brain disorders [21]. However, the exploration of mechanisms underlying the therapeutic benefits from grafted human NPCs has been limited. Currently, it is believed that the potential benefits of grafted NPCs to host brain could be achieved by cellular neuroprotection. Grafted NPCs were found to secret neurotrophins, such as brain-derived neurotrophic factor (BDNF) in the host brain [22, 23]. Secreted BDNF mediates enhancement of hippocampal synaptic density, and finally leads to the improvement of brain functions, such as cognitive ability associated with AD [15, 22, 24].

It is well known that many types of brain disorders exhibit disrupted neural circuits or synaptic networks that are directly related to deficits of brain functions in patients [25]. Therefore, the characterization of grafted human NPCs in terms of their differentiation, maturation and functional integration in host brain would provide more informative clues to elucidate whether and how human grafts could function in a cell replacement manner. We have systematically characterized grafted human iNPCs in the brain of AD mouse, and confirmed that the functionally matured neurons from grafted iNPCs contributed to the reconstruction of neural circuitry in AD brain [3]. In this study, we continuously investigated the possible cellular changes of grafted human iNPCs in the brain of non-human primates. Our data first revealed that grafted human iNPCs differentiated into both neurons and astrocytes in the monkey brain, but displayed a clear bias to neurons (Figs. 1 and 2). Then, we found that human iNPC-derived neurons gradually matured in the monkey brain, and survived well even 1 year after transplantation (Figs. 1 and 3). The gradual decrease of Ki67^ + ^cells within grafted human iNPCs implicated the low proliferative ability of human iNPCs, which is closely associated with the safety issues of cell transplantation (Fig. 2). The long-term survival and limited proliferative ability of donor cells are essentially critical for developing a successful and safe cell therapy.

Functional maturation and synaptic integration of grafted cells is critical for the repair of injured/damaged neural network. Our previous work in mouse found that the grafted cells show a gradual hyperpolarization in the resting membrane potential and a decrease in the input resistance during maturation in the host brain [3]. In this study, we performed whole-cell recording in acute slices from adult monkey brains and measured electrophysiological properties of human iNPC-derived neurons in host basal forebrain (Fig. 4). After 10 months in the host monkey brain, human iNPC-derived neurons possessed membrane properties comparable to mature neurons. The generation of repetitive action potentials and the dependence on input current intensity would ensure the grafted cells to integrate scalable inputs and send outputs accordingly. Except for the neuronal excitability, the spontaneous occurrence of excitatory postsynaptic events indicates the formation of functional synapses onto the grafted cells (Fig. 4). These electrophysiological results suggest that the grafted human iNPC-derived neurons in the monkey brain are mature and integrated to the local neural circuits. Therefore, our findings provide comprehensive characterizations of human NPCs in the brain of non-human primates, which will facilitate the therapeutic application of human NPCs in regenerative medicine.

### Research limitations

The limitations of this study can be interpreted as the impossibility to test the therapeutic potential of human iNPCs in non-human primate models of Alzheimer’s Disease (AD). In our previous study, the therapeutic benefits of the same human iNPC line have been systematically tested and verified in AD mouse [3, 26]. Being a follow-up study, the human iNPC-based cell therapy should be ideally carried out in AD monkeys. Unfortunately, it remains unknown whether the established AD monkeys acquire cognitive deficits associated with AD [27]. Then, we assess the possibility of human iNPCs to serve as donor cells in adult non-human primates in this study first by characterizing the long-term survival and functional maturation of grafted human iNPCs in monkey brains. Additionally, due to the complicated cognitive performance of non-human primates, the measurements of cognitive abilities of monkeys are much more difficult than those of mice, which has actually remained as a central challenge in the field. Then, great efforts will be taken to develop easy and reliable behavioral tasks and platforms that can be used to properly assess the cognitive abilities of non-human primates, including the AD monkeys with and without the grafted human iNPCs in the near future.

## Materials and methods

### Non-human primates

Five wild-type non-human primates (cynomolgus monkeys, *Macaca fascicularis*) aged 9–13 years old were recruited for human iNPCs transplantation in this study. The care of ­non-human primates and experimental procedures involved in this study were thoroughly reviewed and approved by the Animal Care and Use Committee of Wincon Theracells Biotechnologies Co, LTD., in accordance with the Association for Reassessment and Accreditation of Laboratory Animal Care (AAALAC) guideline. During the study, animals were individually housed in stainless steel cages at the primate facility of Wincon Theracells Biotechnologies Co, LTD. in Nanning, Guangxi, China, which is fully accredited by the AAALAC International. Animals were fed twice daily and supplemented with fresh fruits and the miscellaneous enrichments once a day. All animals were maintained on a 12-h light and/or 12-h dark cycle under room temperature at 22°C–28°C with a relative humidity of 30%–75% and water supply ad libitum.

### Human iNPCs transplantation into the brain of non-human primates

The generation of integration-free human iNPCs from human adult peripheral blood mononuclear cells (PB MNCs) was described in our previous studies, which was approved by the Biomedical Research Ethics Committee, SIBS, CAS, and Ruijin Hospital Ethics Committee, Shanghai JiaoTong University School of Medicine, with written informed consent from the donors [3, 26]. With the sequential treatment of five transcription factors (4 Yamanaka factors plus an anti-apoptotic factor BCL-XL) and a cocktail of four chemicals (SB431542, CHIR99021, VPA and Forskolin, SCVF), the immobilized PB MNCs were directly reprogrammed into NPCs in N2B27 medium. The NPC colonies were carefully picked up and successfully passaged to generate iNPC lines possessing similar properties of human native NPCs in terms of, self-renewal ability, multi-potential capacity, typical NPC marker expression patterns and transcriptional profiles [3, 26]. The well-established human iNPCs that were labeled with GFP will serve as donor cells and will be bilaterally delivered into the basal forebrain of cynomolgus monkeys. Briefly, GFP-labeled human iNPCs at passage 15 were cultured into neural sphere. Then, neural spheres were dissociated into single cells using Accutase and suspended in neural differentiation medium. MRI scanning was performed on each primate prior to surgery to identify stereotaxic coordinates. The suspension was diluted to a concentration of 2 × 10^5^ cells/μL and 10 μL of suspension was injected per tract using Hamilton syringe (gauge 22s) by ­MRI-guided stereotaxic surgery combined with convection enhanced delivery system [27]. Before surgery, non-human primates were anesthetized with intramuscular atropine (20 mg/kg), ketamine (10 mg/kg), and sodium pentobarbital (20 mg/kg). Two tracts of injection per side of basal forebrain were performed as shown in Table 1. The precise injection site was guided by MRI parameters. A stereotaxic instrument was used to fix the head of primate during surgery. The needle was pushed into the target brain region at a rate of 1 mm/min through the small hole and held in place for 10 min. Then, 10 μL volume of human iNPCs suspension was injected into the target site of brain at a rate of 1 μL/min. The needle was held in place for 20 min following human iNPCs transplantation and then drawn back at a rate of 1 mm/min. To avoid the immunogenicity of human iNPCs in the ­non-human primate brain, we administrated the ­non-human primates with immunosuppressor cyclosporin A from 2 days before surgery until the day of euthanasia at a dosage of 30 mg/kg tapered to 15 mg/kg.

### Immunostaining

Animals were perfused with saline under deep anesthesia with sodium pentobarbital (30 mg/kg intravenously). Brains were dissected out and coronally cut into 4 mm thick blocks which were then fixed with 4% paraformaldehyde (PFA) for 3 days at 4°Ⅽ. After three washes with PBS, blocks were sequentially immersed in 15% and 30% sucrose solution at 4°Ⅽ for dehydration. Brain blocks were further cryo-sectioned into brain slides of 40 mm thickness using a microtome (Leica SM2000R) and stored in ethylene glycol solutions at ‐20°Ⅽ.

For immunostaining, the brain slides were washed with PBS three times and quenched of endogenous peroxidase activity in PBS containing 0.3% H_2_O_2_ for 30 min. After permeabilization and blocking in the 10× blocking buffer containing 5% donkey normal serum, 1% BSA, and 0.4% Triton X-100 for 2 h at room temperature, brain slides were incubated in primary antibody diluted by 1× blocking buffer for about 40 h at 4°Ⅽ. The primary antibodies used were as follows: monoclonal mouse-anti-STEM121 (TAKARA, Y40410, 1:500), polyclonal ­rabbit-anti-NEUN (Millipore, ABN78, 1:500), polyclonal rabbit-anti-GFAP (Abcam, ab16997, 1:500), monoclonal ­rabbit-anti-Ki67 (Abcam, ab16667, 1:200), monoclonal ­mouse-anti-Tuj1 (BioLegend, 801201, 1:400), monoclonal mouse-anti-MAP2 (Sigma-Aldrich, M4403, 1:200), polyclonal rabbit-anti-NF-H (Proteintech, 18934-1-AP, 1:800), polyclonal rabbit-anti-VGluT1 (GeneTex, GTX133148, 1:200), monoclonal mouse-anti-GAD67 (Millipore, MAB5406IF, 1:200), polyclonal ­goat-anti-ChAT (Millipore, AB144P, 1:100), polyclonal ­rabbit-anti-TH (Millipore, AB152, 1:200), monoclonal ­rabbit-anti-SYNAPTOPHYSIN (Abcam, ab32127, 1:500). After three washes in PBS, brain slides were incubated with ­species-appropriate Alexa secondary antibodies (Jackson Immunoresearch Laboratories) for 2 h at room temperature. To reduce the tissue autofluorescence, brain slides were dipped briefly in distilled water, and then treated with 5 mM CuSO4 in 50 mM ammonium acetate buffer (pH 5.0) for 30 min. DAPI was used to counterstain nuclei. The whole brain images were captured with VS120 Virtual Slide Microscope (Olympus). Confocal images were collected with Leica TCS SP8 STED confocal laser scanning microscope. For quantification, at least five fields were randomly chosen for each primate.

### Electrophysiological recording

Animals were anesthetized with sodium pentobarbital (30 mg/kg intravenously). The brain tissues were immediately dissected out and immersed in ice-cold slicing solution. In ice-cold sucrose-based slicing solution (artificial cerebrospinal fluid (ACSF), listed below but with NaCl replaced with equimolar sucrose) that had been bubbled with 95% O_2_ and 5% CO_2_, tissue blocks containing grafts or hippocampus were sliced coronally with a vibratome (Leica VT1000S). Slices (350 μm thick) were collected and incubated at 35°C in ACSF containing (in mM): NaCl 126, KCl 2.5, MgSO_4_ 2, CaCl_2_ 2, NaHCO_3_ 26, NaH_2_PO_4_ 1.25, and dextrose 25 (315 mOsm, pH 7.4). After 60-min incubation, slices were then incubated at room temperature until whole-cell patch-clamp recording was performed.

Acute slices were transferred to the recording chamber and perfused with aerated ACSF at a rate of 1.2 mL/min. Slices were visualized under upright infrared differential interference contrast microscope (BX51WI, Olympus). The electrical signals were amplified by Multiclamp 700B (Molecular Devices). Voltage and current traces were low-pass filtered at 10 kHz and sampled at 25 kHz using pClamp (Molecular Devices). Grafted cells were identified by their expression of GFP, and Granular cells were identified by their location and morphology. The target cells were recorded with patch pipettes filled with internal solution (in mM): K-gluconate 145, MgCl_2_ 2, Na_2_ATP 2, HEPES 10, and EGTA 0.2 (286 mOsm, pH 7.2). Biocytin (Sigma, B4261) (0.2%) was also added in internal solution for post hoc avidin staining (Avidin-594, Thermofisher, S32355). The grafted GFP + neurons were patched as previously described under room temperature (25°Ⅽ) [3]. The impedances of patch pipettes were 3–5 MΩ, and the data with series resistant higher than 25 MΩ were discarded.

In current clamp mode, step currents (with steps of 10/20 pA, 500 ms in duration) were applied to evoke action potentials (APs). Hyperpolarizing current pulses (‐10 pA, 500 ms) were injected to test the input resistance. In voltage-clamp mode, spontaneous excitatory postsynaptic currents (sEPSCs) were recorded when membrane potentials were clamped at ‐70 mV. The kinetics of PSCs were analyzed with MiniAnalysis 6.03 (SynaptoSoft Inc., NJ, USA).

### Statistical analysis

For immunostaining quantification of cell counting, at least five fields from three brain sections were randomly chosen for each monkey and were quantified manually. The total length of GFAP^ + ^astrocyte fibers and the area of NEUN^ + ^neurons were measured by StrataQuest software version 6.0.1.145 (TissueGnostics, Vienna, Austria). The quantification weas performed on at least six fields from three brain sections for each monkey.

All statistical analyses were performed in GraphPad Prism software (GraphPad 7.0). Cell counting and electrophysiological data were presented as mean ± SEM. One-way ANOVA with Tukey’s multiple comparison post hoc test was used. Statistical significance was set at *P < .05, **P < .01, ****P < .0001.

## Supplementary Material

lnac008_suppl_Supplementary_Figures

lnac008_suppl_Supplementary_Table_S1

lnac008_suppl_Supplementary_Figure_Legends
