# Supplementary figures and images for: The long-term survival and functional maturation of human iNPC-derived neurons in the basal forebrain of cynomolgus monkeys
Source: Life Med. 2022 Jun 28;1(2):196–206. doi: 10.1093/lifemedi/lnac008 (PMC11749281; doi:10.1093/lifemedi/lnac008)

Figure S1

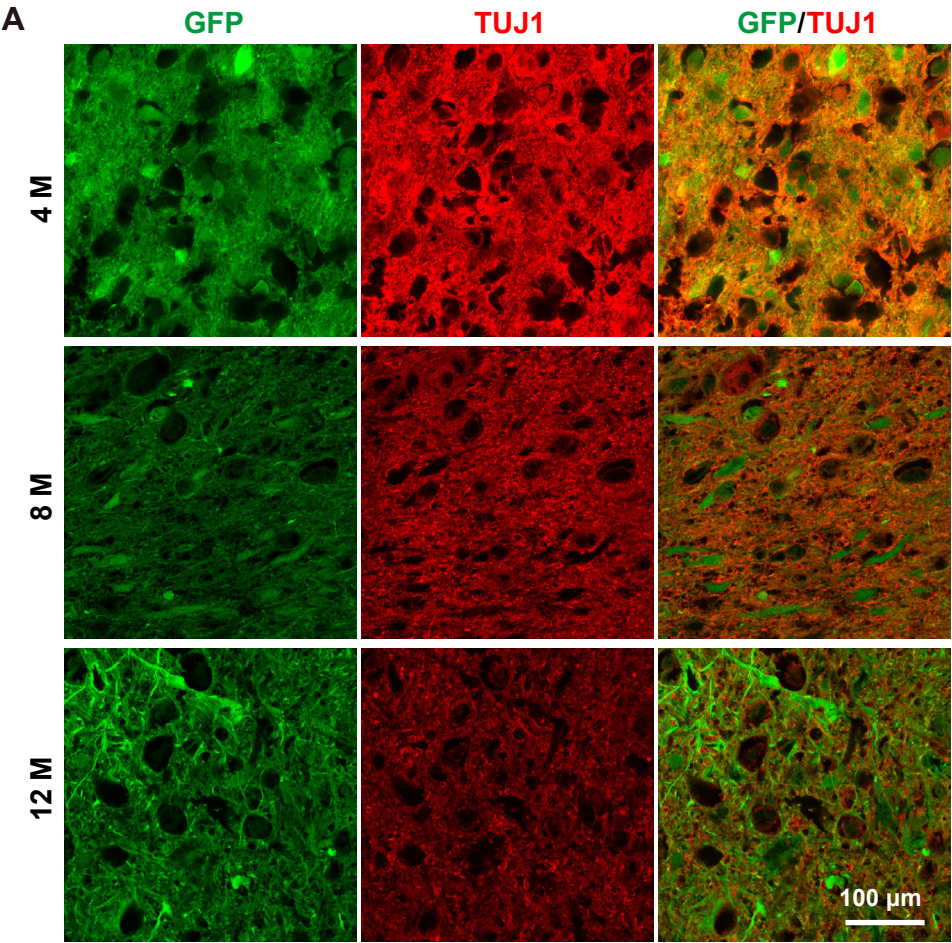

Figure S2

A

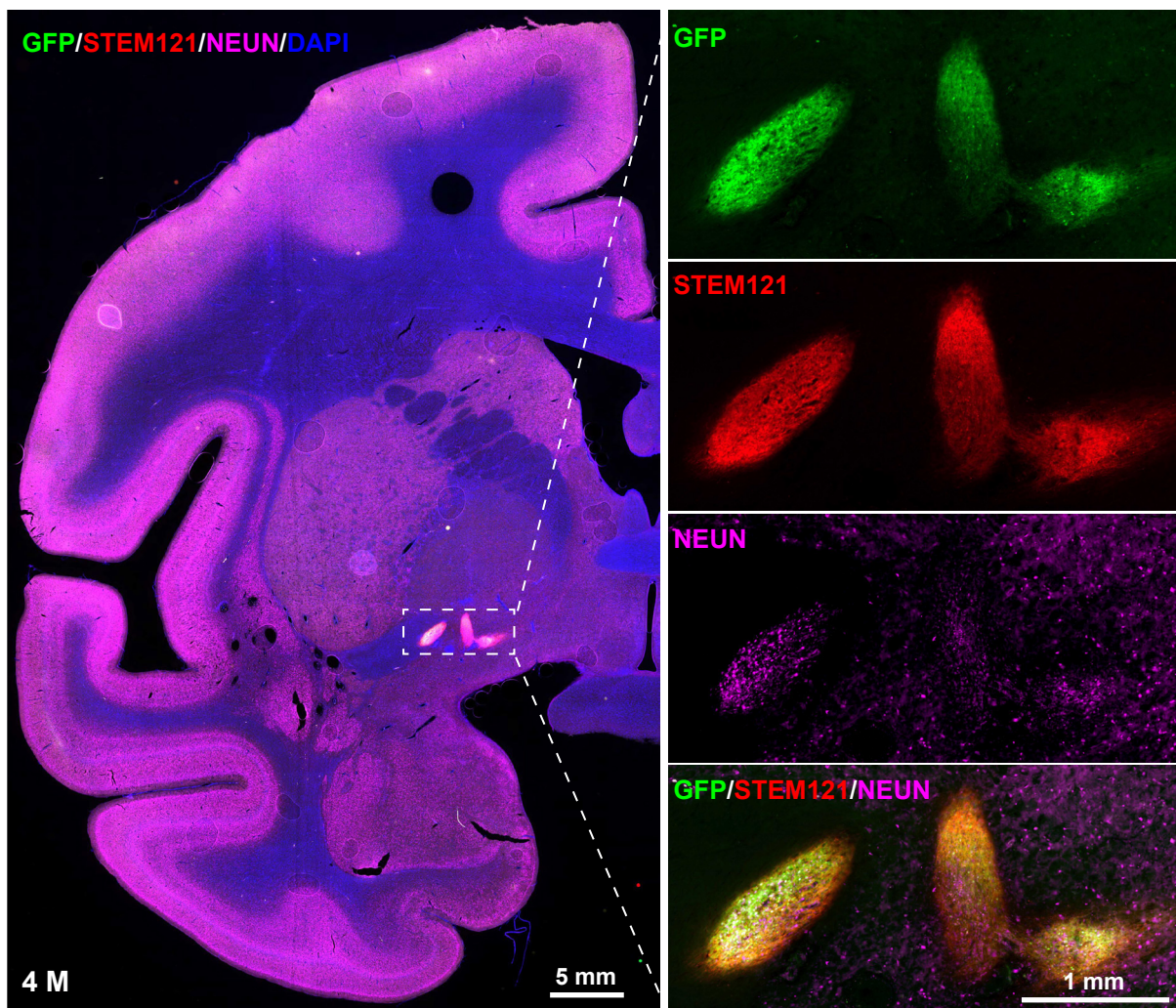

Figure S3

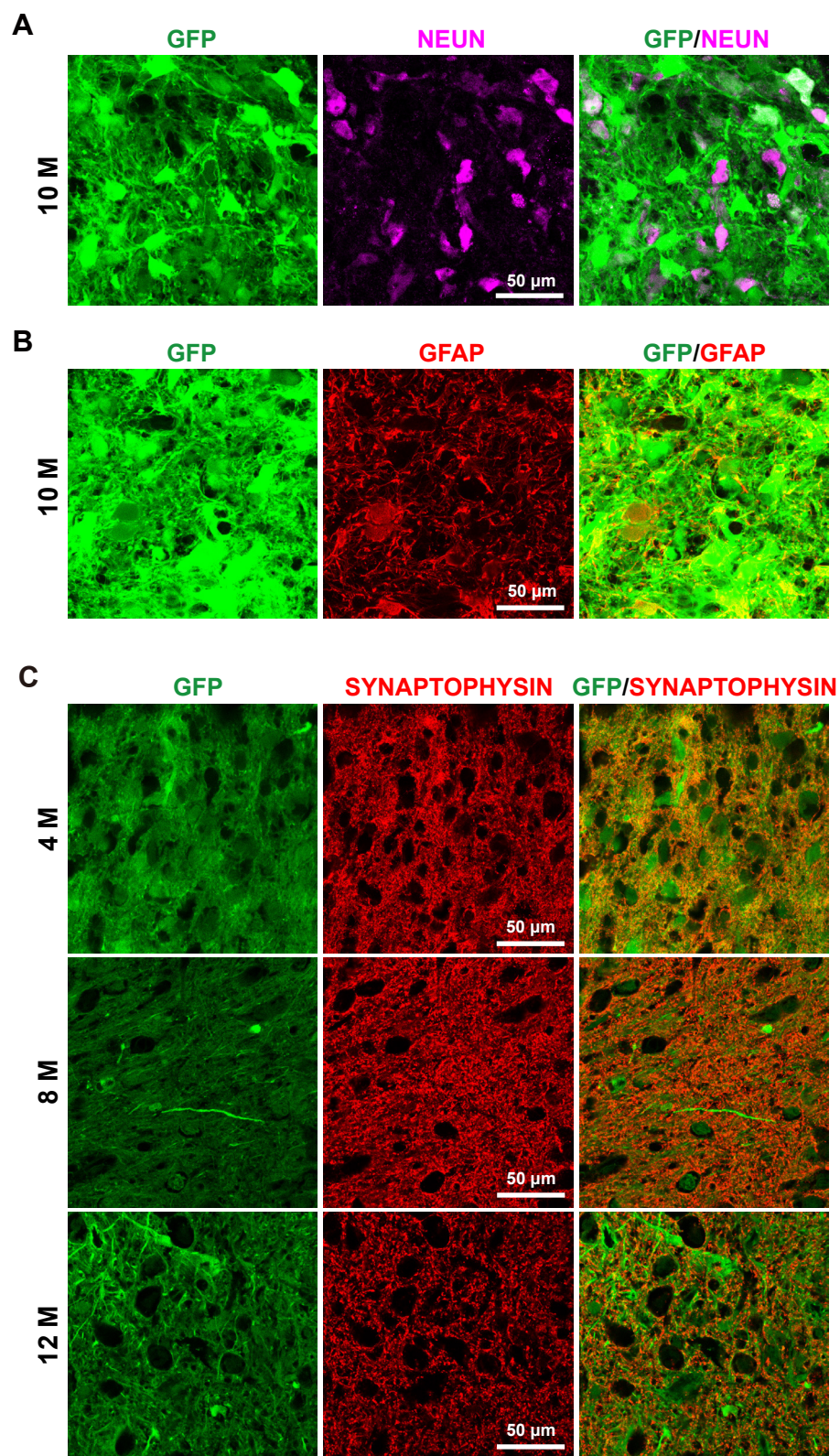

Supplement: lnac008_suppl_Supplementary_Figures [file lnac008_suppl_Supplementary_Figures.pdf]
