# Supplementary material for: The long-term survival and functional maturation of human iNPC-derived neurons in the basal forebrain of cynomolgus monkeys
Source: Life Med. 2022 Jun 28;1(2):196–206. doi: 10.1093/lifemedi/lnac008 (PMC11749281; doi:10.1093/lifemedi/lnac008)
Supplement: lnac008_suppl_Supplementary_Table_S1 [file lnac008_suppl_Supplementary_Table_S1.docx]

**Table S1. Detail information for cell counting and quantification.**

| **Analysis** | **Types of counting/Months post transplantation** | **4 M** | **8 M** | **12 M** |
| --- | --- | --- | --- | --- |
| Counting of GFP^+^ grafted cells | Number of fields counted for GFP^+^ grafted cells | 15 | 14 | 21 |
|  | Number of total GFP^+^ grafted cells counted per field (mean ± SEM) | 71.07 ± 5.448 | 53.56 ± 3.222 | 56.47 ± 2.818 |
| Counting of astrocytes | Number of fields counted for astrocytes | 8 | 9 | 14 |
|  | GFAP^+^/GFP^+^ cells per field (%) (mean ± SEM) | 11.61 ± 0.701 | 9.672 ± 0.699 | 11.34 ± 0.798 |
| Counting of neurons | Number of fields counted for neurons | 7 | 5 | 7 |
|  | NEUN^+^/GFP^+^ cells per field (%) (mean ± SEM) | 48.17 ± 2.232 | 59.86 ± 5.516 | 49.81 ± 4.035 |
| Counting of Ki67^+^ cells | Number of fields counted for Ki67^+^ cells | 8 | 9 | 15 |
|  | Ki67^+^/DAPI^+^ cells per field (%) (mean ± SEM) | 5.91 ± 0.892 | 2.443 ± 0.553 | 1.086 ± 0.359 |
| Measurement of astrocytes | Number of fields for measuring the length of GFAP^+^ astrocyte fibers | 8 | 6 | 11 |
|  | Total length of GFAP^+^ astrocyte fibers per field (μm) (mean ± SEM) | 982.3 ± 55.96 | 1290 ± 88.28 | 2079 ± 60.65 |
| Measurement of neurons | Number of fields for measuring the area of NEUN^+^ neurons | 11 | 6 | 7 |
|  | Mean area of NEUN^+^ neurons per field (μm^2^) (mean ± SEM) | 142.6 ± 6.763 | 200.1 ± 6.242 | 254.3 ± 20.92 |
