# Supplementary material for: The long-term survival and functional maturation of human iNPC-derived neurons in the basal forebrain of cynomolgus monkeys
Source: Life Med. 2022 Jun 28;1(2):196–206. doi: 10.1093/lifemedi/lnac008 (PMC11749281; doi:10.1093/lifemedi/lnac008)
Supplement: lnac008_suppl_Supplementary_Figure_Legends [file lnac008_suppl_Supplementary_Figure_Legends.docx]

Figure S1. The neuronal differentiation of human iNPCs in the monkey brain at different time points after transplantation.

Immunofluorescence analysis of the expression of neuronal marker TUJ1 within human GFP+ grafts 4, 8 and 12 months post transplantation.

Scale bars: 100 μm.

**Figure S2. The survival and neuronal differentiation of human iNPCs in the monkey brain 4 months post transplantation.**

A. Immunofluorescence analysis of human grafts in the basal forebrain of cynomolgus monkey. Immunostaining with NEUN for neurons, with human-specific cytoplasmic marker STEM121 for grafted GFP^+^ human cells. Cell nuclei were counterstained with DAPI. Left panel, the coronal image of hemisphere of monkey with GFP^+^ grafts in the basal forebrain; Right panel, enlarged views of grafts.

Scale bars: 5 mm in left panel; 1mm in right panel.

**Figure S3. The differentiation of human iNPCs in the brain of monkeys 10 months post transplantation and the detection of synapse within human grafts** **4, 8 and 12 months post transplantation.**

A-B. Immunostaining of NEUN and GFAP within GFP^+^ human grafts 10 months post transplantation.

C. Immunostaining of SYNAPTOPHYSIN within GFP^+^ human grafts 4, 8 and 12 months post transplantation.

Scale bars: 50 μm in A-C.
